# Partial Lower Compound Impressions with the Mouth Closed

**Published:** 1919-07

**Authors:** Samuel G. Supplee

**Affiliations:** New York


### PARTIAL LOWER COMPOUND IMPRESSIONS WITH THE MOUTH CLOSED

BY SAMUEL G. SUPPLEE, NEW YORK

The fact that many patients will not wear dentures made to supply missing bicuspids and molars in the lower jaw, has caused the failure of a great many upper dentures which otherwise would have been satisfactory.

Inasmuch as this article is intended to deal only with the impressions and construction of dentures for the lower jaw, we will assume that most of the upper natural teeth are intact or present in the form of an upper denture.

A few moments of careful thought in the right direction will convince the careful observer why patients have experienced difficulty in wearing lower dentures. The answer in a few words is the statement that they have been taking large impressions with the mouth open and the tongue down in the floor of the mouth, and expecting the patient to use a small plate made from this impression when the mouth is closed, the teeth exerting many pounds of pressure and the tongue in contact with the roof of the mouth, where it generally is during both mastication and swallowing.

To convince yourself that there is a difference in the buccal region of the alveolar ridge between no-biting strain and normal-biting pressure, just place the thumb and index finger on the outside of the cheek directly opposite to the region of the molars, the thumb on one side and the finger on the other, and squeeze lightly; first close the teeth lightly, then bite hard, and note the change due to the action of the masseter muscle when biting hard. I have failed to find any one who holds that muscle down when in action.

While these muscular attachments are not connected directly to the crest of the ridge, they are effective indirectly through the medium of the soft tissue and because of this action, plates made from plaster impressions with the mouth open or with the cheek tissues distended, have to be narrowed down so as to give free play to the muscular attachments so that there is practically no saddle to take the stress of mastication.

WHY PLATES HAVE BEEN MADE NARROW IN THE PAST

Consider what must happen when a patient bites down on a plate made from an impression taken with the mouth open. Naturally, as the plate is forced down by the power of occlusion, the muscle must raise as the result of the same impulse, and creates a scissor-like motion, so that either the tissue will be cut or the pain be so great that the patient can not bite hard.

By taking the impression of the tissues and muscles when the mouth is closed and under biting strain, we can construct a denture that will extend out on these tissues and muscles, fitting best when the greatest pressure is being applied, and increasing the bearing surface from 50 per cent. to 200 per cent. without any discomfort to the patient.

A plate made from an impression taken under biting strain will fit best when maximum pressure is applied, but it will not fit as well when the pressure is released.

In fact, I have made many partial lower plates that appeared to rock from side to side when testing them with the mouth open, yet patients have found them perfectly stable when masticating.

The writer has been a strong advocate of compound as the best means of taking impressions and has published so many articles to emphasize the importance of understanding the working consistency of compound and the necessity of having proper heating facilities, that it is needless to repeat. These refer only to full denture technic for which the consistency of the material and the observance of detail in technic is very exacting, whereas in partials much of the detail is eliminated and the consistency of material is not of such vital importance. But he would render the best service himself with the proper compound heating apparatus, whereby he can maintain a definite water temperature of about 150 to 160 degrees.

### BASIC IMPRESSIONS AND FACE PIECE

The impression consists of two distinct parts, namely, the base impression and the face piece.

The base impression includes the lingual surface and the saddle area to be covered by the denture.

The face piece is laid on later to complete the impression of the buccal or labial surface of the standing teeth or gum, to guide in selecting or setting up teeth.

This face piece is secured by placing the hardened base impression in the mouth, and laying a small roll of softened compound over the buccal border of the exposed teeth or gums.

By taking impressions in this way, we immediately place a great majority of our cases in the no-undercut class, which is simple to handle, for a great majority of the undercuts exist bucco-lingually and do not interfere with the removal of the basic impression.

The material can be prepared and placed in the mouth so that the buccal or labial border of the teeth will be exposed and left till it sets stone hard. Then it can be removed without danger of distortion or breaking. If a very slight undercut exists, the teeth are free to move sufficiently to permit the removal of the impression. The face piece takes care of the apparently difficult part of the impression.

### DIAGNOSING THE CASE

Your success in handling this method will be determined greatly by your skill in *diagnosing your case,* and planning how you are going to remove and replace the finished denture, before taking your impression.

You should observe these plans very carefully in taking out your base impression and replacing it in position.

In other words, the side having the least undercut should be removed first, and the side having the least undercut should be put back in position first.

### THE IMPRESSION TRAY

All Supplee partial trays are designed on the plan of a flat occlusal plane, and can be secured from the dental depots. They come with the complete set of Supplee trays, or in sets of eight partial trays. They are made of soft aluminum, so that the tray can be placed between the miscellaneous teeth, and the patient can bite it into shape or at least bend it sufficiently so that the tray will be held firmly in position between the teeth while the base impression is being muscle-trimmed and the material is setting.

The object of the tray is to serve merely as a carrier. It is illustrated, for convenience sake, on an articulator, so that you can see the position the tray assumes in the mouth.

### ADJUSTING THE TRAY

Fit your tray regardless of the buccal or labial portion of the teeth or gums, excepting to see that it does not touch the ridge or extend out into the cheek or tissues so as to distend them in any way.

Have the patient close on the tray, pressing hard enough to bend it so that it can be held firmly without pain, due to pressure of the tray on the tissues, or strain on the teeth.

The No. 6, Partial Lower Supplee Tray, is designed to take all cases where the bicuspids and molars are missing, and the No. 5 is used in cases where the molar may be present.

### PREPARING THE MATERIAL

Place the material in the tray so that it will approximate the form of the *proposed plate*, without any regard for the buccal or labial surface of the standing teeth, using sufficient quantity to fill the space between the tray and the ridges.

It should be warm throughout, but that portion next to the tray should be slightly cooler than the portion to come in contact with the tissue.

The preparation of the material is the most important part of the process, and should be very deliberate.

### FASTENING COMPOUND TO THE TRAY

To insure the compound adhering so that it will not leave the tray when you are taking the impression out of the mouth, the tray should be thoroughly dried and the surface of the compound should be passed over the flame until it is sizzling hot before placing it in contact with the dry metal.

Cool the compound next to the tray by immersing the tray in cold water after the compound is placed on it and shaped to the approximate formation; then pass the surface that is to come in contact with the teeth and tissues over the flame to soften it to a flowing state. Dip this surface in hot water before placing the compound in the mouth, and have the patient bite it to position. You have thus established three strata of compound of different consistency and temperature, which is very advantageous.

### TAKING THE IMPRESSION

In taking the impression, place the tray and the material in the mouth in the same way as you propose to insert the finished denture, carrying the tray three-quarters of the way to position and then have the patient close and drive it the rest of the way. Caution the patient to hold the jaws firmly closed, then swallow twice, give the lip and cheek movements and then continue to hold firmly while you lift the lips and quickly force all material away from the buccal or labial side of standing teeth, using the finger or an instrument.

Sufficient pressure should be brought to bear over the cheeks to reduce the compound to the desired fullness while in a flowing state.

After the excess is removed and the buccal and labial surfaces exposed, have the patient give the lip and cheek movement a second time and massage lightly; then let the compound set till it is stone hard, being very careful not to permit the patient to open until this is accomplished.

*Ice water or a cold air syringe always should be used before removing the impression.*

### EXPOSE THE BUCCAL SURFACE OF THE TEETH

The Supplee tray is designed especially for this work, and is so formed that you can push the compound to the edge of the tray with the index finger, cutting the excess away in a scissor-like manner, while the compound is still warm.

If the teeth are not exposed so as to permit the impression being readily removed, let the compound set until it is sufficiently hard so that you can just dent it with the finger nail. Then it is very simple to use a lance or the small blade of a pen knife to cut the compound away before it becomes stone hard.

*Caution:* The patient must keep the teeth closed firmly on the back of the tray during this operation.

If care has been exercised to see that no compound has covered the buccal or labial surface of the standing teeth, there will be no "drag" to the compound, particularly if you are careful in removing the impression to carry it *back* first before raising it.

To insure against this, when the basic impression is removed from the mouth, it is wise to dip the impression in cold water for about two seconds and quickly place the impression back in the mouth and press firmly into position with finger pressure.

### EQUALIZE BITING STRAIN BEFORE CORRECTING THE IMPRESSION

If there are defects in the impression, due to lack of material they can be corrected readily with the tracing stick.

*Caution:* Before correcting a partial impression, it is absolutely essential that you make sure that the patient has not closed forward or created a muscle-strained condition when driving the impression to position. Otherwise you will have difficulties in making corrections should they be required. To insure against this, it is wise to trace sufficient compound on the back of a compound biting block to indicate that patient closes each time in the same place.

To appreciate the importance of this, one must thoroughly understand the principles outlined under the title of ''Muscle Strain,'' published in one of the dental journals some time ago.

### FACE PIECE COMPLETES THE IMPRESSION

The face piece never should be made until the corrections to the base impressions are complete, when it takes but a few minutes to place the impression back in the mouth; have the patient close and lay a small roll of compound under the lip or cheek opposite the buccal or labial border of standing teeth, then draw the lip over it and press on the outside with the finger.

This will conform the compound over the buccal and labial surfaces and make a nice joint with the rest of the compound impression. Let this cool thoroughly. When removing the impression, ignore this face piece by taking hold of the handle of the tray or some part of the basic impression and remove it in the same manner you would the finished denture.

In the majority of cases, the face piece will remain in position or open up sufficiently to let the impression pass over the bell-shaped portion of the teeth.

As soon as you can take the face piece out of the mouth, it should be laid back to place on the impression, so that if slightly bent it readily can be pressed back to position. The pressure should be brought to bear directly over the right-angled points that were cut for the purpose of making a clean and definite point. If ice water is used, there will be no danger of its bending, as the compound will break clean before it will bend.

### DESIRABLE CASTS

Casts made from these impressions will present the perfect contour of all standing teeth, to guide in selecting or setting up teeth, and as much of the exact lingual formation as possible to maintain and still insert the plate without filing or fitting.

The additional time expended in taking an impression of this kind will be offset by the time that otherwise would have been expended in adjustment of a denture made from a plaster impression.

In making dentures from impressions taken by this technic, it is possible for us to make the denture cover the entire saddle area indicated by the muscle trimming without any discomfort to the patient, and in the majority of cases, it would be from two to five times the area possible to obtain when an impression has been taken by the old-fashioned plaster technic.

The extent to which this rule will apply to the lingual portion will depend a great deal on the consistency of the material and attention to details in technic, a matter into which the writer has not attempted to enter because of the limitations of space devoted to this paper, but those who would like to know more of the details for partial upper as well as the partial lower, can procure same in booklet form.—*Dental Summary.*

